# Characterization of a Hyperthermostable Alkaline Lipase from* Bacillus sonorensis* 4R

**DOI:** 10.1155/2016/4170684

**Published:** 2016-01-21

**Authors:** Hemlata Bhosale, Uzma Shaheen, Tukaram Kadam

**Affiliations:** DST-FIST Sponsored School of Life Sciences, Swami Ramanand Teerth Marathwada University, Nanded 431606, India

## Abstract

Hyperthermostable alkaline lipase from* Bacillus sonorensis* 4R was purified and characterized. The enzyme production was carried out at 80°C and 9.0 pH in glucose-tween inorganic salt broth under static conditions for 96 h. Lipase was purified by anion exchange chromatography by 12.15 fold with a yield of 1.98%. The molecular weight of lipase was found to be 21.87 KDa by SDS-PAGE. The enzyme activity was optimal at 80°C with *t*_1/2_ of 150 min and at 90°C, 100°C, 110°C, and 120°C; the respective values were 121.59 min, 90.01 min, 70.01 min, and 50 min. The enzyme was highly activated by Mg and *t*_1/2_ values at 80°C were increased from 150 min to 180 min when magnesium and mannitol were added in combination. The activation energy calculated from Arrhenius plot was 31.102 KJ/mol. At 80–120°C, values of Δ*H* and Δ*G* were in the range of 28.16–27.83 KJ/mol and 102.79 KJ/mol to 111.66 KJ/mol, respectively. Lipase activity was highest at 9.0 pH and stable for 2 hours at this pH at 80°C. Pretreatment of lipase with MgSO_4_ and CaSO_4_ stimulated enzyme activity by 249.94% and 30.2%, respectively. The enzyme activity was greatly reduced by CoCl_2_, CdCl_2_, HgCl_2_, CuCl_2_, Pb(NO_3_)_2_, PMSF, orlistat, oleic acid, iodine, EDTA, and urea.

## 1. Introduction

Hyperthermophiles are the group of organisms growing at temperatures between 80 and 110°C. This group is represented by bacterial and archeal species found in all types of terrestrial and marine hot environments. The hyperthermophilic enzymes or thermozymes derived from these organisms exhibit extreme thermostability and highest activity at temperatures above 70°C [1], some being highly active at and above 110°C [2]. Hence, such enzymes are used as model systems for enzyme based research including enzyme research, molecular basis of thermostability, and deciding the upper temperature limit for enzyme function. Thermozymes are extremely stable and active at high temperatures and offer many biotechnological advantages over mesophilic enzymes such as easier purification by heat treatment, higher resistance to chemical denaturants, and reduced risk of microbial contamination [1]. They also offer high reaction rates and process yields by lowering viscosity, causing increased diffusion rates and substrate availability and maintaining favorable equilibrium with endothermal reactions [3]. Thermozymes isolated from hyperthermophiles growing at the temperature range of 80–110°C are expected to be more thermostable than their mesophilic correspondents as these organisms are in full harmony with the existing thermal conditions and expected to secrete the enzymes that are completely stable at these temperatures to support their physiological processes [4].

Lipases are the most important group of industrial biocatalysts that can be applied both as hydrolases and as synthetases and proved their enormous potential in various biotechnological applications. The unique characters of lipases such as high stability in organic solvents, their broad substrate specificity, and high enantioselectivity greatly increased their demand in industrial market. The current market scenario of hydrolytic enzymes positioned lipases at the top third rank after proteases and amylases and their annual market is targeted to reach about 590.5 million dollars by 2020 [5]. However, most of the industrial processes operate at relatively high temperature and alkaline pH conditions. Hence, the thermoalkalostability of lipases is one of the desired characteristics to endure harsh processing conditions used during industrial applications. Thermostable lipases are the need of food, cosmetics, detergent, and pharmaceutical industries [6, 7].

Most of the research on lipases is concentrated on isolating highly thermostable lipases from different thermophilic microbial sources. While thermostable lipases are known to be produced by* Bacillus* sp. which have unique protein sequence and inherent biochemical properties, lipases from* Burkholderia ambifaria* YCJ01 [8],* Aneurinibacillus thermoaerophilus strain HZ* [9], and* Pseudomonas* sp. [10] are also reported. However, the studies related to thermoalkalostable lipases from hyperthermoalkalophilic organisms are scanty. In the present study we are reporting for the first time the isolation and identification of highly thermostable lipase producing hyperthermophilic strain of* Bacillus sonorensis* 4R. Purification of enzyme and its thermodynamic and biochemical properties are also reported.

## 2. Materials and Methods

### 2.1. Materials

All media ingredients, diethylaminoethyl-cellulose (DEAE-cellulose), phenylmethylsulfonyl fluoride (PMSF), and bovine serum albumin (BSA), were purchased from HiMedia. All other chemicals used were of analytical grade.

### 2.2. The Lipolytic Organism and Enzyme Production

The lipolytic strain of* Bacillus sonorensis* 4R used in the present study was isolated from Thar Desert ecosystem of Jaisalmer, Rajasthan, India (lat. 27′00N and 71°00E). The strain was grown on alkaline tributyrin inorganic salt agar (g/L: K_2_HPO_4_, 1; MgSO_4_, 1; NaCl, 1; ammonium sulphate, 2; CaCO_3_, 2; FeSO_4_, 0.001; MnCl_2_, 0.001; ZnCl_2_, 0.001; and tributyrin, 10 mL) at pH 9.0, 80°C for 7 days. Lipase production was carried out by growing active culture of 4R (5%) in 2 L glucose-tween inorganic salt broth (g/L: K_2_HPO_4_, 1; MgSO_4_, 1; NaCl, 1; ammonium sulphate, 2; CaCO_3_, 2; FeSO_4_, 0.001; MnCl_2_, 0.001; ZnCl_2_, 0.001; glucose 10, CaSO_4_, 100 mM; and tween-80, 10 mL) adjusted to pH 9.0. The flasks were incubated at 80°C for 4 days under static conditions. At the end of incubation, the culture broth was centrifuged at 10,000 rpm for 30 min at 4°C to obtain cell-free supernatant. The supernatant was used as crude source of* Bacillus sonorensis* lipase (BSL) and analyzed for lipase activity and protein content.

### 2.3. Identification of Lipolytic Organism

Bacterial genomic DNA was isolated using geneO-spin Microbial DNA Isolation Kit (geneOmbio Technologies, Pune, India). This DNA was used as template for PCR analysis using the primers 27F: 5′-AGAGTTTGATCMTGGCTCAG-3′ and 1492R: 5′-TACCTTGTTACGACTT-3′. The amplification conditions were 95°C for 10 min, 57°C for 1 min, 72°C for 90 sec, and final amplification at 72°C for 10 min. The PCR products were purified by using a geneO-spin PCR Product Purification Kit (geneOmbio Technologies, Pune, India) and were directly sequenced using an ABI PRISM BigDye Terminator V3.1 Kit (Applied Biosystems, USA). The sequences were analyzed using Sequencing Analysis 5.2 software. BLAST analysis was performed at BlastN site at NCBI server (http://www.ncbi.nlm.nih.gov/BLAST) and evolutionary relationship of 4R was deduced by constructing phylogenetic tree.

### 2.4. Lipase Assay and Protein Determination

The assay was performed by using the modified method described by Selvam et al. [11] based on olive oil hydrolysis. To the reaction mixture containing 1 mL of tris-HCl buffer (pH 9.0), 2.5 mL of deionized water, and 3 mL of olive oil emulsion (10% gum arabic emulsified with 5% olive oil), 1 mL of crude enzyme for test and 1 mL of deionized water for blank were added in separate tubes. The reaction mixture was mixed thoroughly by swirling and incubated at 80°C for 30 min. After incubation, enzyme substrate reaction was terminated by addition of 3 mL of 95% ethanol and mixed by swirling. The amount of fatty acids liberated due to lipase activity was estimated by titrating the contents of assay mixture against 0.05 M NaOH using thymolphthalein as a pH indicator. The end point observed was from colorless to light blue. One unit of lipase was defined as the amount of enzyme required to release 1 *μ*mole of fatty acid under assay conditions. Protein content of all fractions was determined by Bradford assay [12] by using BSA as a standard protein.

### 2.5. BSL Purification

The crude lipase was purified using a two-step procedure including ammonium sulphate precipitation followed by dialysis and DEAE-cellulose ion exchange chromatography. The cell-free supernatant was pretreated at 80°C for 30 min to eliminate the appearance of additional proteins and brought to 80% saturation by adding finely powdered ammonium sulphate. The flask was kept overnight at 4°C and the precipitate was collected by centrifugation at 10,000 rpm for 20 min at 4°C. The precipitate was dissolved in phosphate buffer (0.1 M, pH 9.0) and dialyzed overnight against the same buffer.

The desalted enzyme obtained from dialysis step was loaded on chromatography column (1.5 × 15 cm) packed with DEAE-cellulose and preequilibrated with 0.1 M phosphate buffer (pH 9.0). The enzyme was eluted with linear gradient of NaCl (0.1–0.5 M) in phosphate buffer. The flow rate of column was adjusted to 0.5 mL/min and protein concentration (280 nm, UV Vis Shimadzu) and lipase activity of eluted fractions were determined as mentioned before after desalting.

### 2.6. Determination of Molecular Mass of BSL

The molecular mass of purified BSL was determined by sodium dodecyl sulphate polyacrylamide gel electrophoresis (SDS-PAGE) technique using HiPer SDS-PAGE Kit (HiMedia) according to the manufacturer's instructions. A broad range of unstained protein standards (insulin [3.5 kda], aprotinin [6.5 kda], lysozyme [14.3 kda], soya bean trypsin inhibitor [20.1 kda], carbonic anhydrase [29.0 kda], ovalbumin [43.0 kda], BSA [66.0 kda], phosphorylase [97.4 kda], and myosin [205.0 kda]) was used as molecular mass makers. The gel was stained with 0.025% Coomassie Brilliant Blue R-250 staining solution provided in the kit and destained overnight by adding 7% acetic acid solution. The molecular mass of purified BSL was determined from a plot between log⁡MW and relative migration values (*R*_*f*_) of standard protein markers. The activity of purified fraction obtained after electrophoresis was confirmed by zymogram analysis. The gel was prepared by supplementing 1% tributyrin; the sample was loaded and subjected to electrophoresis. The gel was stained and destained as mentioned before and the location of band on gel was observed for presence of clear zone due to tributyrin hydrolysis.

### 2.7. Effect of Temperature on BSL Activity and Stability

The optimum temperature for BSL activity was determined over the temperature range of 80–120°C (80, 90, 100, 110, and 120°C) by preincubating aliquots of purified lipase in phosphate buffer (100 mM, pH 9.0) at respective temperatures for 30 min. After incubation, the fractions were cooled on ice and assayed for BSL activity. To determine the effect of temperature on enzyme stability, different aliquots of purified enzyme were preincubated separately at 80–120°C for 3 h in phosphate buffer (100 mM, pH 9.0) and residual activity was measured at intervals of 30 min.

### 2.8. Effect of Divalent Cations and Polyols on Thermostability of BSL

The effect of varying concentrations of CaSO_4_ and MgSO_4_ and different polyols including glycerol (3C), ethylene glycol (5C), inositol (5C), sorbitol (5C), and mannitol (6C) on thermal stability of BSL was studied by preincubating various enzyme fractions in presence of respective compounds at 80°C for 3 h. The aliquots were withdrawn after every 30 min, ice-cooled, and used for residual activity determination. The activity was compared with initial lipase activity observed before incubation in presence of Ca, Mg, and polyols. The polyol showing improved thermostability was selected over the range of 20–100 mM for further study at 80°C. Similarly, cumulative effect of selected polyol (60 mM) and MgSO_4_ (80 mM) on thermostability of BSL was determined.

### 2.9. Thermodynamic Parameters

The thermodynamic parameters related to BSL activity at elevated temperatures (80–120°C) were determined in terms of half-life (*t*_1/2_), denaturation constant (*K*_*d*_), enthalpy of denaturation (Δ*H*), free energy of denaturation (Δ*G*), and entropy of denaturation (Δ*S*). The inactivation rate constants were calculated from a plot of residual activity versus time and used for estimating half-lives. The activation energy of thermal inactivation (*E*_*a*_) was determined from the Arrhenius plot between ln⁡*K*_*d*_ and 1/*T* (*K*) as described before [13]. The values of Δ*H*, Δ*G*, and Δ*S* for inactivation were calculated according to the following equations, respectively, as described by Gummadi [14]:

(1) ΔH=Ea−RT

(2) ΔG=−RTln⁡Kd·hKb·T

(3) ΔS=ΔH−ΔGT,

where *R* = 8.314 JK^−1^ mol^−1^ is the universal gas constant, *T* is absolute temperature, *h* is Plank's constant, and *K*_*b*_ is Boltzmann's constant.

### 2.10. Effect of pH on BSL Activity and Stability

To determine the effect of pH on BSL activity, the aliquots of enzyme were preincubated in buffers of different pH values (sodium phosphate, 0.1 M, pH 7.5–8; tris-HCl, 0.1 M, 8.5–9.0; carbonate-bicarbonate, 0.1 M, pH 9.5–10.5; sodium phosphate-NaOH, 0.1 M, pH 11-12) for 30 min at 80°C. After incubation the fractions were ice cooled and enzyme activity was determined under assay conditions. To determine the effects of pH on stability, aliquots of BSL were preincubated with buffer of pH 9.0 for 180 min at 80°C and the residual activity was determined at intervals of 20 min.

### 2.11. Effect of Metal Ions on BSL Activity

The effects of various metal ions, namely, Ca^++^, Mg^++^, Cu^++^, Pb^++^, Co^++^, Cd^++^, and Hg^++^, as CaSO_4_, MgSO_4_, CuCl_2_, Pb(NO_3_)_2_, CoCl_2_, and HgCl_2_ on BSL activity were studied. The aliquots of BSL (10 *μ*L) were preincubated in presence of different metal ion concentrations (25–150 mM) at 80°C for 30 min and subjected to lipase assay. The effect of metal ions on BSL activity was determined by comparing the enzyme activities in absence of these compounds.

### 2.12. Effect of Chemical Modulators on BSL Activity

The effect of different chemical modulators on BSL activity was tested by preincubation of properly diluted enzyme at 80°C for 30 min in presence of selected chemical modulators. The chemical modulators (ethylene diamine tetra-acetic acid (EDTA), urea, PMSF, iodine, orlistat, and oleic acid) were set at 5 mM and after preincubation, BSL activity was determined under assay conditions. The effect of chemical modulators on BSL activity was determined by comparing the enzyme activities in absence of these compounds.

## 3. Results and Discussion

### 3.1. Growth and Lipase Production by* Bacillus sonorensis* 4R

**Figure 1:**
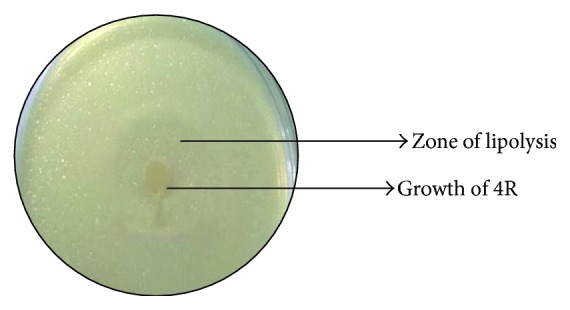
Lipase producing bacterial isolate* Bacillus sonorensis* 4R: colonies on tributyrin agar showing zone of lipolysis after 4 days of incubation at 80°C and pH 9.0.

The lipase producing* Bacillus sonorensis* 4R, isolated from soils of Thar Desert area in Jaisalmer, Rajasthan, India, was detected using tributyrin agar plates. The isolate showed good tributyrin hydrolysis efficiency on plates (28 mm) (Figure 1) as well as in broth (51.33 U/mL) after 4 days of incubation at 80°C. The hyperthermoalkalophilic bacteria optimally grow within temperature range of 80–110°C and are found in all terrestrial and marine hot environments. The selected soil sample of Thar Desert after enrichment in inorganic salt medium supplemented with 1% tributyrin at 80°C and pH 9.0 successfully isolated potential lipase producing thermoalkalophilic strain of 4R.

**Figure 2:**
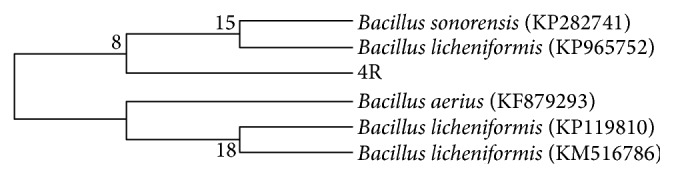
Neighbor joining tree based on 16S gene sequencing showing phylogenetic relationship between* Bacillus sonorensis* 4R and related members of the genus* Bacillus*.

The isolate was identified as* Bacillus sonorensis* on the basis of its morphological characteristics and 16S rRNA sequencing. The 4R strain has ability to grow at temperature between 80 and 100°C and 8.0–11.0 pH with optimum growth at 80°C and pH 9.0. It appeared as a facultative anaerobe, Gram-positive long rod, nonmotile, catalase positive bacterium and had the capacity to reduce nitrate and produced acid from glucose, arabinose, xylose, and mannitol. The 1286-base-pair sequence obtained by 16S rRNA sequencing has been deposited to NCBI gene bank database with accession number* KT 368092*.* Bacillus sonorensis* 4R shared the highest homology of 100% with* Bacillus sonorensis *strain ZJY-537. From the phylogenetic analysis it was confirmed that 4R was closely associated with* Bacillus sonorensis* which is a close lineage of* Bacillus licheniformis* and other members of genus* Bacillus* (Figure 2). Hence, the strain 4R was identified as* Bacillus sonorensis*. So far,* Bacillus sonorensis* was reported to be isolated from Sonoran Desert [15] and Kalbadevi estuary, Mumbai [16]. Abundance of thermophilic* Bacillus* species including* Bacillus licheniformis*,* Bacillus aerius*,* Bacillus sonorensis*,* Bacillus subtilis*, and* Bacillus amyloliquefaciens* in hot springs, salt marshes, and desert soil of Morocco has been observed by Aanniz et al. [17]. The study also highlighted the growth of isolated* Bacillus* species at temperature range between 30 and 80°C. All species of* Bacillus sonorensis* showed good growth from 30 to 55°C whereas none of the isolates grew above 70°C. Different studies have reported the dominance of strains of* Bacillus* species in various geothermal habitats including Japanese desert [18] and Atacama Desert soil [19] with special reference of common occurrence of* Bacillus sonorensis* in deserts of Morocco [15]. However, the hyperthermophilic growth at 80°C and above temperatures and lipase production capacity of* Bacillus sonorensis* were not reported earlier. Only few reports on sonorensin, a food preservative [20], and lipopeptide antibiotic production [21] by strain of* Bacillus sonorensis* are available. In this context, we are reporting for the first time the isolation of hyperthermophilic lipase producing* Bacillus sonorensis* from Thar Desert of Rajasthan.

### 3.2. Purification of BSL

The lipase produced by* Bacillus sonorensis* was purified by using a sequential procedure including salt precipitation, desalting by dialysis, and chromatography on DEAE-cellulose column. The results of the lipase purification profile are summarized in Table 1. The enzyme was finally purified 12.15-fold over crude extract with 1.98% recovery.

**Figure 3:**
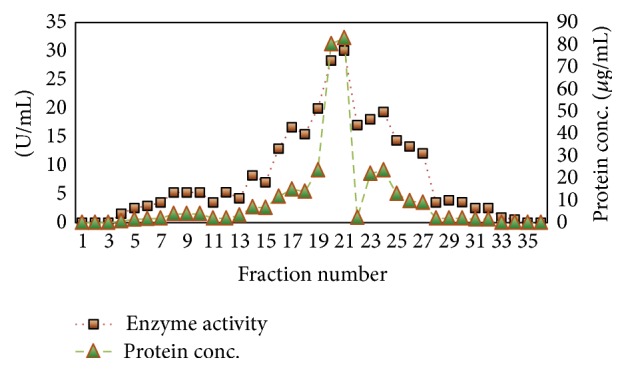
Elution profile of BSL for purification on DEAE-cellulose column.

**Figure 4:**
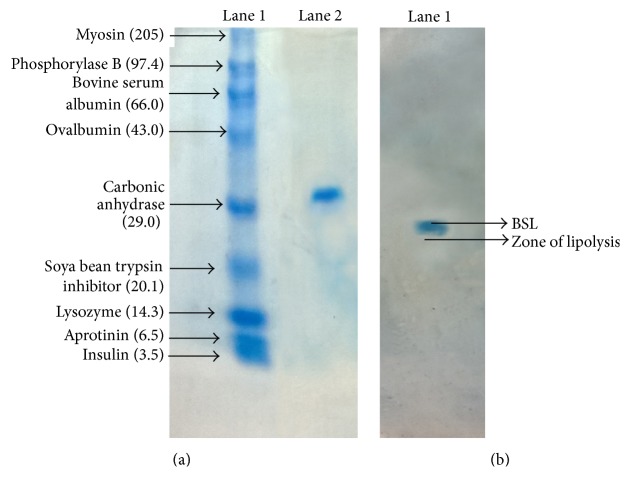
SDS-PAGE of hyperthermostable lipase from* Bacillus sonorensis* 4R. (a) Lane 1: standard protein molecular mass markers, Lane 2: purified BSL. (b) Activity characterization of BSL by zymogram analysis.

Chromatography of lipase on DEAE-cellulose ion exchange column resulted in one prominent peak at the 21st fraction (Figure 3). The active fractions were pooled and the homogeneity of purified enzyme was confirmed by the presence of a single band corresponding to an apparent molecular mass of 21.87 KDa on SDS-PAGE gel (Figure 4(a)). Lipase activity in the purified band was checked by observing presence of lipolysis zone in gels supplemented with 1% tributyrin (Figure 4(b)).

### 3.3. Effect of Temperature on BSL Activity

**Figure 5:**
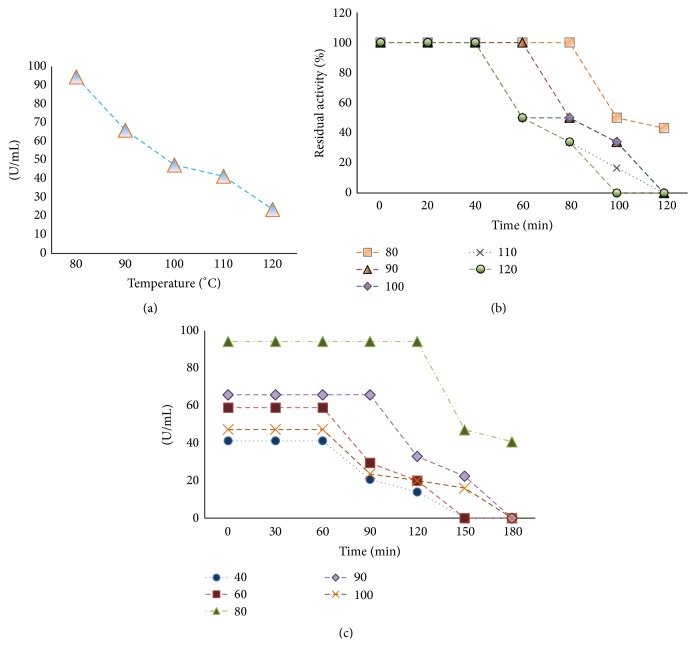
(a) Effect of temperature on BSL activity. (b) Thermostability of BSL at temperatures from 80 to 120°C. (c) Thermal activation of BSL at elevated temperatures.

The effect of different temperatures on activity of purified lipase is shown in Figure 5(a).* Bacillus sonorensis* produced lipase was more active in temperature range of 80–120°C with more than 50% of its original activity remaining above 90°C up to 120°C after 30 min exposure (Figure 5(b)). The optimum temperature recorded for the lipase activity of TM12350, a recombinant lipase from a hyperthermophilic bacterium* Thermotoga maritima*, was 70°C [22] with maximum activity retained for 60 min at 70°C while maintaining more than its 50% activity within 8 h. At higher temperature the confirmation of enzyme is disrupted which results in reduced affinity sites for substrate [23]. Hence, in the present study when the temperature was increased from 80 to 120°C, a gradual decrease in catalytic activity of BSL was observed. However, the degree by which the activity was decreased was not convincing as BSL retained more than 50% of its original activity at 120°C. The lipase exhibited significant stability at 80°C with a half-life (*t*_1/2_) of 150 min whereas the values of *t*_1/2_ reduced to 121.59 min, 90.01 min, 70.01 min, and 50 min, respectively, at 90°C and above temperatures (100°C, 110°C, and 120°C) and at pH 9.0. These characteristics indicated that BSL is a highly thermostable lipase retaining about 50% activity at and above 100°C. This study for the first time showed the highly thermostable nature of lipase produced among* Bacillus* family and probably among all reported lipases. For lipase produced by* Bacillus licheniformis* MTCC6824, [24] reported *t*_1/2_ values of 82 min, 75 min, and 48 min at 45°C, 50°C, and 55°C whereas Shariff et al. [25] showed thermoactive nature of L_2_ lipase at a temperature range of 55–80°C with temperature optima at 70°C and *t*_1/2_ of 2 h at 60°C. However, the reported thermostability at alkaline pH (9.0) in the present study was higher where enzyme was retaining its 50% activity at and above 100°C. Lipases at high temperature and alkaline pH are of immense importance in food industry and pharmaceuticals due to their process conditions operating at high temperature (45–50°C) and pH (8.0). The thermostability exhibited by BSL was greater than other thermostable lipases, such as lipase from* Bacillus* species SP42 with *t*_1/2_ of 45 min at 70°C [26], esterase from* Thermoanaerobacter* sp. with *t*_1/2_ of 90 min at 70°C [27], and lipase from the hyperthermophilic* Aneurinibacillus thermoaerophilus-HZ* with half-life of 80 min at 70°C. The enzymes activated at and above 40°C are said to undergone thermal activation [10]. In the present study the BSL was only activated at high temperatures (80°C) and the activity was very poor at 40°C indicating thermal activation of BSL (Figure 5(c)). The characteristic of an enzyme to show thermal activation depends on the hydrophobic amino acid content of the protein and lipases are known to be rich in hydrophobic amino acids [28, 29]. Hence, it is expected that the thermal activation of BSL in the present study might be contributed by its hydrophobic amino acids content.

### 3.4. Effect of Divalent Cations and Polyols on BSL Activity

**Figure 6:**
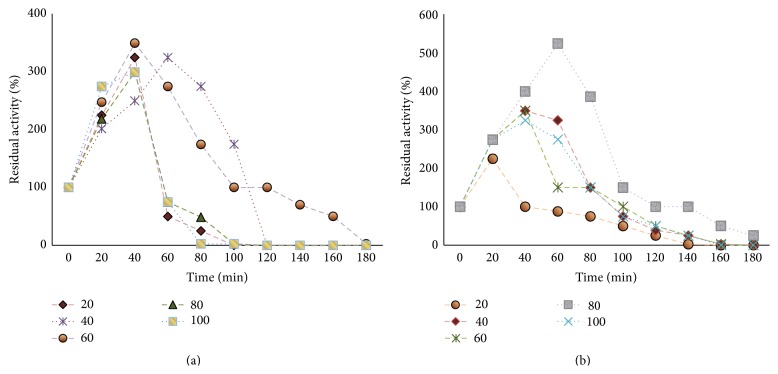
(a) Effect of CaSO_4_ (20–100 mM) on BSL thermostability. (b) Effect of MgSO_4_ (20–100 mM) on BSL thermostability.

The catalytic activity of BSL was greatly increased over control at 80°C in presence of Ca^2+^ and Mg^2+^ at all concentrations (20–100 mM). CaSO_4_ when used at 60 mM and 80 mM concentrations caused 249.08% and 199% respective enhancement in BSL activity whereas, with increase in incubation time, the activity was gradually decreased and reported absent after 3 h incubation at all concentrations (Figure 6(a)). BSL activity was also found to increase significantly in presence of MgSO_4_ (80 mM) after 20–100 min exposure. The highest increase in activity was observed after 60 min incubation (423.6%) and thereafter, the activity was slowly reduced (Figure 6(b)).

**Figure 7:**
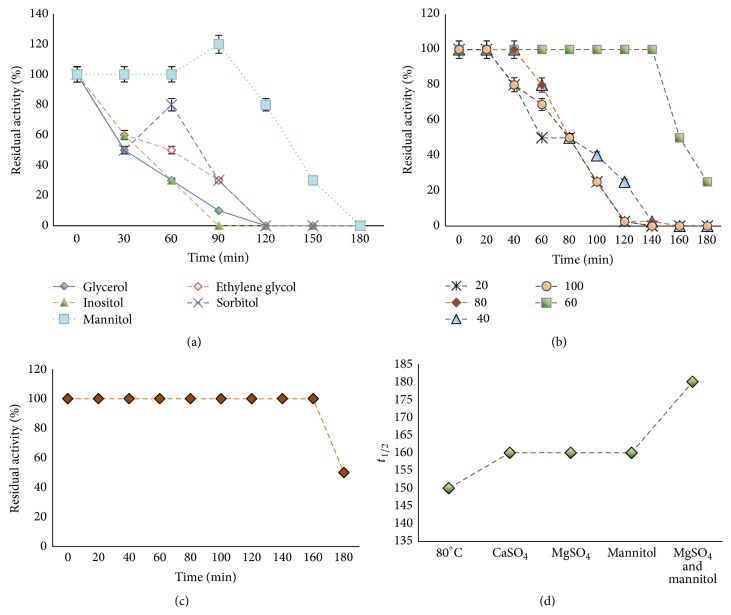
(a) Effect of different polyols (50 mM) on BSL activity. (b) Effect of mannitol (20–100 mM) on BSL thermostability. (c) Cumulative effect of MgSO_4_ (80 mM) and mannitol (60 mM) on BSL thermostability. (d) *t*_1/2_ values of BSL at 80°C, CaSO_4_ (60 mM), MgSO_4_ (80 mM), mannitol (60 mM), and MgSO_4_ (80 mM) and mannitol (60 mM).

It has been reported earlier that the molecular size and number of hydroxyl groups per molecule of polyol play an important role in mediating the protection against thermal inactivation [30]. In this study mannitol appeared as the best thermoprotectant at 50 mM concentration as observed in terms of approximately 20% enhancement in residual activity after 90 min exposure while retaining 100% activity when incubated for 60 min (Figure 7(a)). However, with further rise in incubation time, the activity was gradually reduced. The effect of different mannitol concentrations on thermostability of BSL was also evaluated. Increasing mannitol concentration up to 60 mM improved the thermostability of BSL with 49.99% of the original activity remaining after 140 min at 80°C. At higher concentration of mannitol (100 mM) reduced thermostability was observed where 100% of residual activity was retained only for 60 min (Figure 7(b)). Addition of polyols can prevent conformational changes of the enzyme by promoting formation of numerous hydrogen bond or salt bridges between amino acid residues, making the enzyme molecule more rigid and, hence, more resistant to the thermal unfolding [31, 32]. However, the selection of the suitable additive depends on the nature of enzyme and it varies from one enzyme to another. Addition of polyols improves thermostability of lipase from* Bacillus licheniformis* MTCC6824 [24], xylanases from* Trichoderma reesei* QM9414 [33], and xylanase from* A. pullulans* CBS135684 [34]. The effect of sorbitol on thermostability of lipase has been identified in* Bacillus licheniformis* MTCC6824 [24]. The cumulative effect of MgSO_4_ (80 mM) and mannitol (60 mM) on thermostability is shown in Figure 7(c). BSL incubated with a combination of MgSO_4_ and mannitol induced a synergistic effect observed in terms of 100% residual activity of BSL remained after 160 min, as compared to 49.99% when incubated with Mg^++^ or mannitol alone. After 3 h, Mg^++^ and mannitol combination was found to retain approximately 50% of original activity as compared to approximately 25% in presence of Mg^++^ or mannitol alone. The *t*_1/2_ values at 80°C were increased from 150 min to 180 min when magnesium and mannitol were added in combination (Figure 7(d)). The loss of enzyme activity at elevated temperature ranges is related to changes in enzyme conformation [30, 35]. Improved thermostability of BSL due to Mg^++^ and mannitol, a higher polyhydric alcohol, might be due to hindered denaturation of catalytic site of enzyme caused by hydration resulting in charge rearrangement and ion complexation. Ion complexation of metal ions such as Ca^++^ is a process with favorable entropy factor that helps in stabilization of enzymes at high temperatures. The role of Ca^++^ ions in maintenance of stable and active enzyme structure is well stated [24]. However, the Mg and mannitol dependent improvement of hyperthermostable lipases is not reported earlier.

### 3.5. Thermodynamic Characteristics

**Figure 8:**
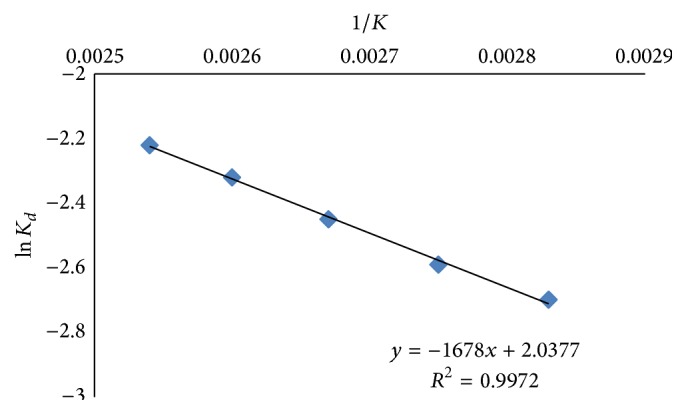
Arrhenius plot for determination of the activation energy of BSL.

The effects of varying temperatures on kinetic and thermodynamic characteristics of BSL are summarized in Table 2. The parameters including half-life period, denaturation constant, entropy, enthalpy, and free energy change at different temperatures were determined from Arrhenius plot as shown in Figure 8. The activation energy calculated from Arrhenius plot was 31.102 KJ/mol at 80°C; the *t*_1/2_ value was 150 min and it appeared to reduce to 121.59 min at 90°C. Further, increase in temperatures reduced half-lives of BSL to 90.01 min, 70.01 min, and 50 min appearing at 100°C, 110°C, and 120°C, respectively. The values of denaturation constant were increased with increase in temperature from 80 to 120°C. The values of Δ*H* were not changed significantly from 80 to 120°C and recorded in the range of 28.16–27.83 KJ/mol. Δ*G* was increased from 102.79 KJ/mol at 80°C to 111.66 KJ/mol at 120°C. It was reported previously that thermodynamically stable proteins exhibit high Δ*G* [36]. The appearing high values of free energy change in denaturation in the present study confirmed the thermodynamic stability of BSL and its better resistance against thermal unfolding conferred at elevated temperatures. This was further supported by observed entropy values. The change in entropy was not noticeable from 80 to 120°C demonstrating nominal changes in enzyme architecture during thermal unfolding.

### 3.6. Effect of pH on BSL Activity and Stability

**Figure 9:**
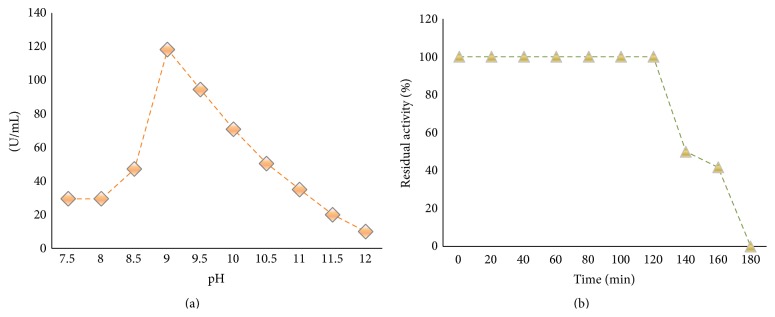
(a) Effect of pH on BSL activity at 80°C. BSL activity at pH 9.0 was set as 100%. (b) Stability of BSL at pH 9.0. BSL activity without preincubation was set as 100%.

BSL exhibited good activity over the pH range 7.5–11 (Figure 9(a)). The maximum activity was observed at pH 9.0 (118.20 U/mL) followed by pH 9.5 (94.4 U/mL) and pH 10 (70.8 U/mL). A rapid decline in the enzyme activity was observed beyond pH 10.0 whereas the activity was lowest at pH 12 (10 U/mL). At pH 9.0 BSL retained its 100% activity for 120 min (Figure 9(b)) followed by a sequential decrease in original enzyme activity from 140 min (49.99%), 160 min (41.68%), and 180 min (0%) exposure. Similarly, Jeagar et al. [25] reported maximum lipolytic activity of L_2_ lipase towards olive oil as a substrate retaining 50% of its original activity at pH 10.0.

### 3.7. Effect of Metal Ions on BSL Activity

**Figure 10:**
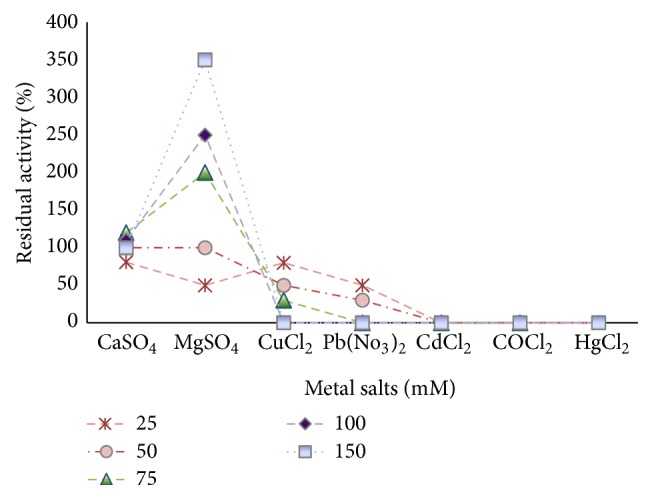
Effect of metal ions on BSL activity. BSL activity without amendment of metal ions was set as 100%.

The effects of varying concentrations of metal ions (25–150 mM) including CaSO_4_, MgSO_4_, CuCl_2_, Pb(NO_3_)_2_, CoCl_2_, CdCl_2_, and HgCl_2_ on BSL activity are shown in Figure 10. A concentration dependent enhancement in lipase activity was found in presence of MgSO_4_. The residual activity was gradually increased from 100% to 349.94% with increase in MgSO_4_ concentration from 50 mM to 150 mM, respectively. Addition of CaSO_4_ at concentration range of 50–150 mM either retained 100% residual activity or caused a marginal enhancement in BSL activity. The activity was reduced drastically in presence of low concentrations of CuCl_2_ (25–75 mM) and Pb(NO_3_)_2_ (25–50 mM) whereas, at higher concentrations (100–150 mM), BSL was completely inhibited. The inhibition of BSL by Cu^++^, a transition metal ion, might be due to the changes brought by metal ion in solubility and behavior of ionized fatty acid at the interfaces affecting the catalytic properties of enzyme [37]. At all concentrations of CoCl_2_, CdCl_2_, and HgCl_2_, BSL lost its 100% original activity indicating highly potent inhibitory nature of these metals.

### 3.8. Effect of Chemical Modulators on BSL Activity

Table 3 shows the effect of different chemical modulators H_2_O_2_, PMSF, EDTA, bile salts, orlistat, and oleic acid at 5 mM conc. on BSL activity. Among the different compounds tested, BSL activity was greatly reduced by 83.33% in presence of PMSF indicating it as a member of serine family. The inhibition occurring in presence of PMSF might be due to modification of essential serine residue, inducing a direct or indirect change in enzyme confirmation [38, 39]. A considerable inhibition in BSL activity was recorded in presence of EDTA (50.01%) and urea (50.01%). Inhibition in presence of EDTA indicated that the BSL is metalloenzyme [40]. Oleic acid is an end product of olive oil hydrolysis mediated by lipase action. BSL activity was reduced by 80% when enzyme was preincubated in presence of oleic acid. The drastic reduction of BSL activity in presence of oleic acid might be due to the end product inhibition, a regulatory process in which the metabolite formed in downstream reactions inhibits the activities of upstream enzyme [41].

## 4. Conclusion

In the present study, a highly thermostable alkaline lipase from a desert isolate* Bacillus sonorensis* 4R was isolated and characterized. On the basis of results, BSL can be used as a potential candidate in various industrial and biotechnological sectors with special mention as additives in detergents and food industries, environmental bioremediations, and molecular biology. However, further works relating to improvement in enzyme yield and other kinetic aspects of enzyme activity are required to understand the catalytic properties of this enzyme. Statistical approach based optimization of BSL production and structural elucidation of lipase is in progress.

## Figures and Tables

**Table 1:** Purification summary of hyperthermostable lipase from *B. sonorensis *4R.

	Protein content (mg/mL)	Total activity	Specific activity (U/mg)	Purification fold	Yield (%)
Crude	0.290	153990	177	1	100
Ammonium sulphate precipitation	0.252	13275	351	1.98	8.62
Dialysis	0.143	5901.5	825	4.67	3.83
DEAE-cellulose column	0.355	3055.96	2152.08	12.15	1.98

**Table 2:** Kinetic and thermodynamic parameters for thermal denaturation of BSL.

Temperature (°C)	*t*_1/2_	*K*_*d*_	Δ*H* {enthalpy (KJ/mol)}	Δ*G* {free energy (KJ/mol)}	Δ*S* {entropy (J/mol/K)}
80	150	46*∗*10^−4^	28.16	102.79	−0.211
90	121.59	57*∗*10^−4^	28.08	105.12	−0.212
100	90	77*∗*10^−4^	28.00	110.28	−0.220
110	70	99*∗*10^−4^	27.91	109.32	−0.212
120	50	138*∗*10^−4^	27.83	111.66	−0.211

**Table 3:** Effect of chemical modulators on BSL activity. Residual activity of BSL was determined by comparing activities before incubation of BSL in presence of modulators.

Chemical modulators	Residual activity (%)
EDTA	49.99
Urea	49.99
PMSF	16.67
Iodine	20
Orlistat	20
Oleic acid	20
